# Single use bioreactors for the clinical production of monoclonal antibodies – a study to analyze the performance of a CHO cell line and the quality of the produced monoclonal antibody

**DOI:** 10.1186/1753-6561-5-S8-P103

**Published:** 2011-11-22

**Authors:** Sonja Diekmann, Constanze Dürr, Alexander Herrmann, Ingo Lindner, Daniela Jozic

**Affiliations:** 1 Roche Diagnostics GmbH, Pharma Biotech Penzberg, 82377 Penzberg, Germany

## Background

In recent years, the use of disposables in the pharmaceutical industry has increased extensively. Disposables can be used in many areas of biopharmaceutical production. The use of disposables not only reduces investment costs, but also requires less manpower to operate, since time consuming change-over procedures are significantly reduced. In addition, disposables enable a high flexibility by reducing unit operation times such as cleaning and sterilization as well as the validation of these procedures [1]. Disposable bioreactors can be subdivided into two main groups, static and dynamic systems. The dynamic systems differ with regard to the power input in stirred, vibromixed or wave-induced systems [2]. Since the power input, the mixing time, the tip speed and the oxygen transfer coefficient may influence the cell culture process and the product quality itself, a thorough characterization of the system used is necessary [3]. SSB (stainless steel bioreactors) and SUB (single use bioreactors) differ in terms of their physical design. Usually, an SSB is equipped with two or three stirrer blades. In contrast, stirred SUBs usually have just one stirrer blade. In addition, the design and the position of the stirrer differ significantly. These distinctions lead to different physical characteristics regarding the power input, mixing time, and tip speed. The SUBs are characterized by a significantly lower power input and tip speed and a significantly higher mixing time. In order to maintain a sufficient oxygen transfer coefficient, the single use bioreactor is equipped with a micro sparger with a pore size of 25 µm. Furthermore, pure oxygen can be used to achieve higher dissolved oxygen concentrations in the cell cultivation medium. This study describes the influence of these technical differences on the performance of a CHO cell line and the product quality of a monoclonal antibody.

## Results

After inoculation, the cells were cultured in both systems in a fed batch mode with two continuous feeds for twelve days. Figure 1a shows the viable cell density as a function of time. Whereas the overall growth characteristic of the cells in both systems are comparable over the whole cultivation time, from 92 h until 188 h, the cells in the SSB are characterized by a significantly (*) faster growth (p< 0.05). The viability of the cells in both systems remains between 90 % and 100 % over the whole cultivation time (data not shown). Figure 1b shows the LDH activity in both systems as a function of time as an indicator of cell lysis. Up to 68 h cultivation time the LDH activity in both systems is comparable. Thereafter, the LDH activity measured in the SUB is significantly (*) higher in comparison to the SSB (p < 0.05). Figure 1c shows the product titer [%] produced by the cells in the SSB in comparison to the SUB and Figure 1d shows the specific productivity. Between 140 h and 250 h the product titer in the SSB is slightly higher in comparison to the SUB, whereas the titer at harvest is comparable. Since the growth of the cells in the SSB is faster in comparison to the cells in the SUB, the specific productivity of the cells cultivated in the SUB is higher. Figure 1d shows the IEC data of the antibody produced by the CHO cells in the SUB and the SSB. Whereas the acidic region of the antibody of the SSB is slightly higher in comparison to the antibody of the SUB the difference is not significant. The main peaks are comparable. The basic region of the antibody of the SSB is slightly lower in comparison to the SUB, balancing the slight increase in acidic region observed for the antibody derived from the SSB.

**Figure 1a:**
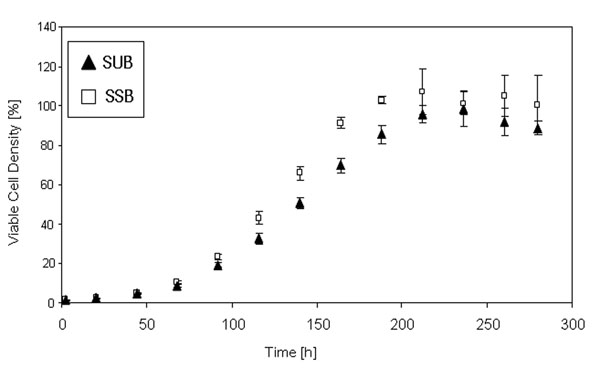
Viable cell density

**Figure 1b:**
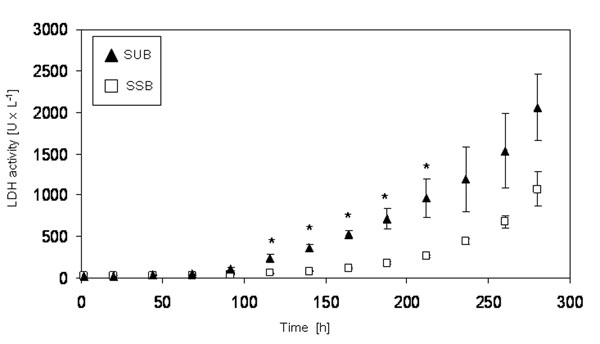
LDH activity

**Figure 1c:**
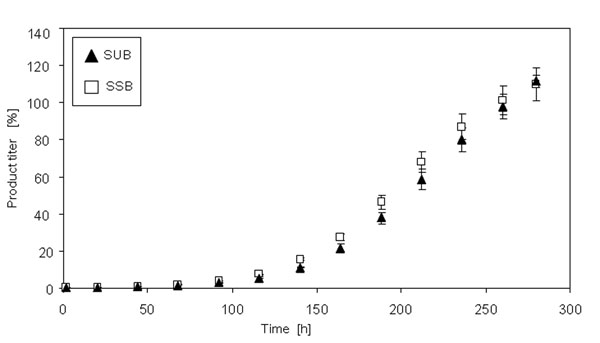
Product titer

**Figure 1d:**
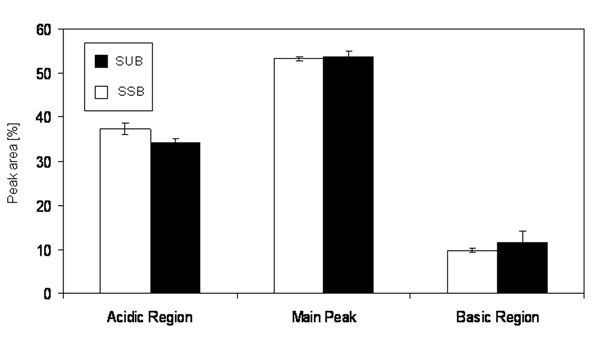
IEC pattern

The SEC patterns of both products are almost identical (Tab. 1). The glycopatterns of the mAB produced in the SUB and of the mAB produced in the SSB shows no significant differences (Fig. 1e). The G0 fraction of the mAB produced in the SSB is slightly higher in comparison to the G0 fraction of the mAB produced in the SUB whereas the G1 fraction of the mAB produced in the SSB is slightly lower compared the mAB produced in the SUB. The G2 fraction is very similar. The G0-Fucose value of the mAB produced in the SSB is higher than for mAB produced in the SUB, which leads to a higher overall a-fucose value for the SSB derived product compared to the SUB derived product. However, all these differences are not significant. All other fractions are comparable between both antibodies. To investigate the influence of both bioreactor types on the impurity profile, the DNA and HCP values of the harvested supernatant were compared. Figure 1f shows the specific DNA concentration (DNA concentration divided by viable cell density) of the harvested supernatant of the SSB and the SUB. The specific DNA concentration of the harvested supernatant of the SSB is slightly higher compared to the SUB. However, theses differences are not significant. Figure 1g shows the specific HCP concentration measured in the harvested supernatant of the SSB and the SUB. The specific HCP concentration (HCP concentration divided by viable cell density) of the SUB is slightly higher compared to the specific HCP concentration of the SSB. Again, these differences are not significant.

**Table 1:** SEC pattern

Bioreactor-type	Monomer [%]	HMW [%]	LMW [%]
SSB	99.7 ± 0	0.267 ± 0.047	0.1 ± 0

SUB	99.7 ± 0	0.3 ± 0	0.1 ± 0

**Figure 1e:**
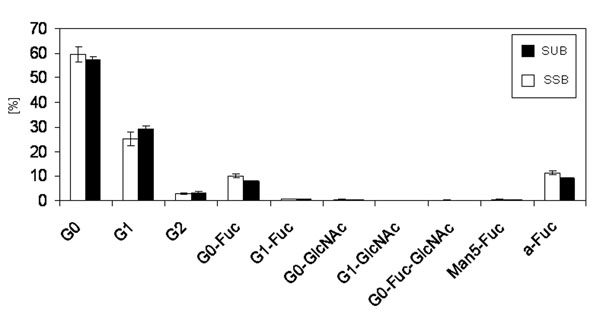
Glycopattern

**Figure 1f:**
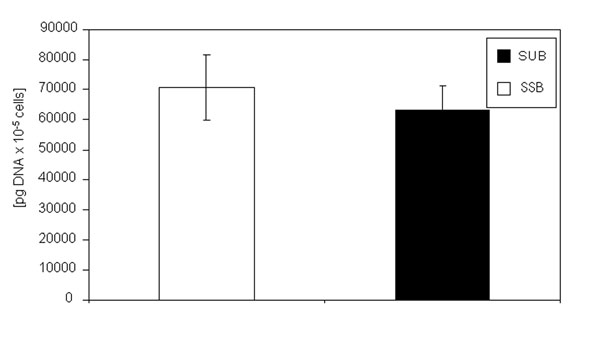
DNA concentration

**Figure 1g:**
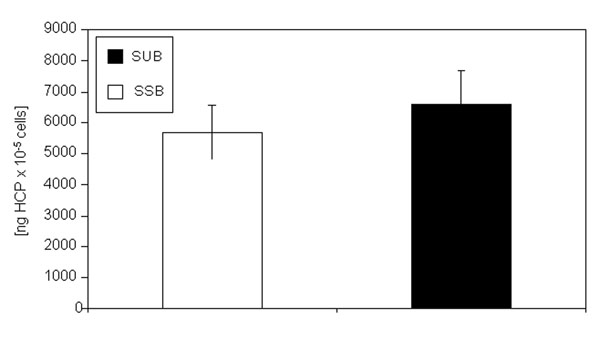
HCP concentration

## Conclusions

The present study describes the influence of a single use bioreactor on the performance of a production CHO cell line, on the product quality of the produced antibody and the process related impurities in comparison to a commercial stainless steel bioreactor. It seems that the microsparger of the SUB lead to a cell damage, which is measured by LDH activity. Nevertheless, our findings indicate that this cell damage has no influence on the productivity, the concentration of DNA and HCP and, most importantly on the quality of the antibody.
